# Supplementary material for: The interactome of intact mitochondria by cross-linking mass spectrometry provides evidence for coexisting respiratory supercomplexes
Source: Mol Cell Proteomics. 2017 Dec 8;17(2):216–32. doi: 10.1074/mcp.RA117.000470 (PMC5795388; doi:10.1074/mcp.RA117.000470)
Supplement: Supplemental Data [file supp_RA117.000470_133922_0_supp_23978_fzffwf.zip › spectra_annotation/mito_spectra_annotation/124-1-4-1-4-1.pdf]

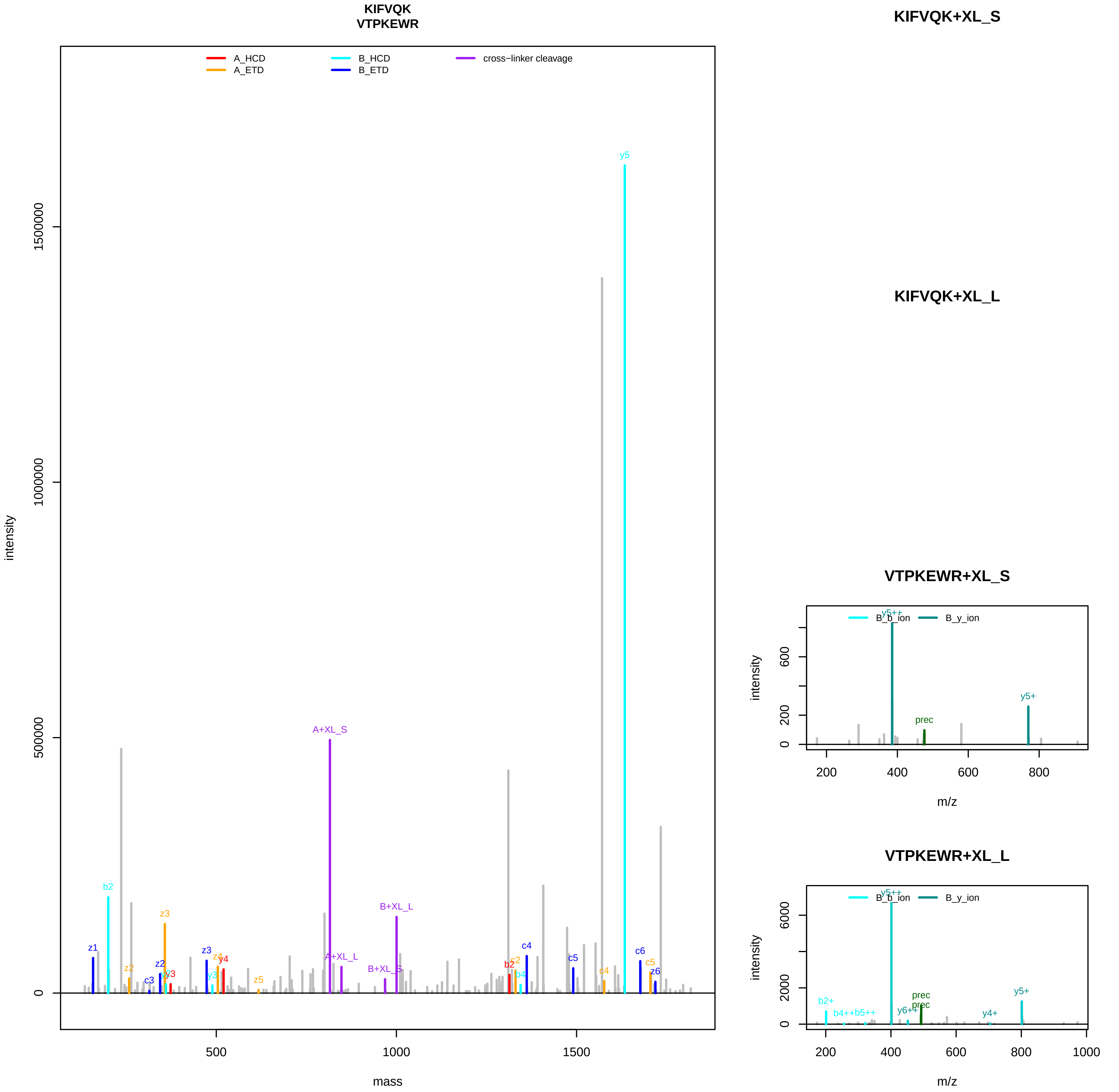
KIFVQK
VTPKEWR
A_HCD
A_ETD
B_HCD
B_ETD
cross−linker cleavage
intensity
mass
0
500000
1000000
1500000
500
1000
1500
y5
A+XL_S
B+XL_L
A+XL_L
B+XL_S
b2
z3
z1
z2
c3
y2
y3
z4
y4
z5
b2
c2
b4
c4
c5
c4
c6
c5
z6
KIFVQK+XL_S
KIFVQK+XL_L
VTPKEWR+XL_S
B_b_ion
B_y_ion
y5++
prec
y5+
m/z
0
200
600
400
800
VTPKEWR+XL_L
b2+
b4++b5++
y6++
prec
y4+
2000
6000
1000
